# Transcriptomics in Rare Minnow (*Gobiocypris rarus*) towards Attenuated and Virulent Grass Carp Reovirus Genotype II Infection

**DOI:** 10.3390/ani13111870

**Published:** 2023-06-04

**Authors:** Jie Ma, Chen Xu, Nan Jiang, Yan Meng, Yong Zhou, Mingyang Xue, Wenzhi Liu, Yiqun Li, Yuding Fan

**Affiliations:** 1 Yangtze River Fisheries Research Institute, Chinese Academy of Fishery Sciences, Wuhan 430223, China; majie_shou@163.com (J.M.); xuchen@yfi.ac.cn (C.X.); jn851027@yfi.ac.cn (N.J.); mengy@yfi.ac.cn (Y.M.); zhouy@yfi.ac.cn (Y.Z.); xmy@yfi.ac.cn (M.X.); liuwenzhialisa@yfi.ac.cn (W.L.); liyq@yfi.ac.cn (Y.L.); 2 National Demonstration Center for Experimental Fisheries Science Education, Shanghai Ocean University, Shanghai 201306, China

**Keywords:** transcriptomics, grass carp reovirus, rare minnow, *Gobiocypris rarus*, spleen, liver

## Abstract

**Simple Summary:**

Grass carp reovirus genotype Ⅱ (GCRV Ⅱ) is the leading cause of death in grass carp. To investigate the involved molecular responses against the GCRV Ⅱ infection, we performed comparative transcriptomic analysis in the spleen and liver of rare minnow injected with virulent and attenuated strains. Results showed that the virulent strain infection especially induced tissue-specific alteration and caused severe suppression of hemorrhage related pathways in spleen. Our finding provides new insights on the interactions between host and GCRV Ⅱ.

**Abstract:**

Grass carp reovirus genotype Ⅱ (GCRV Ⅱ) causes a variety of fish hemorrhagic disease, which seriously affects the sustainable development of grass carp aquaculture in China. Rare minnow (*Gobiocypris rarus*) is an ideal model fish to study the pathogenesis of GCRV Ⅱ. To investigate the involved molecular responses against the GCRV Ⅱ infection, we performed comparative transcriptomic analysis in the spleen and liver of rare minnow injected with virulent strain DY197 and attenuated strain QJ205. Results showed that the virulent DY197 strain induced more differently expressed genes (DEGs) than the attenuated QJ205 strain, and tissue-specific responses were induced. In the spleen, the attenuated and virulent strains induced different DEGs; the attenuated QJ205 infection activated steroid synthesis pathway that involved in membrane formation; however, virulent DY197 infection activated innate immunity and apoptosis related pathways while suppressing cell proliferation and migration related pathways that are important for damage tissue repair, as well as hemorrhage related pathways. In the liver, the attenuated and virulent strains infection induced similar DEGs; both strains infection activated immunity and apoptosis related pathways but suppressed metabolism-related pathways; virulent DY197 infection especially activated protein digestion and absorption-related pathways and suppressed steroid synthesis pathway. To conclude, virulent strain infection especially induced tissue-specific alterations and caused severe suppression of hemorrhage-related pathways in spleen. Our findings will contribute to better understanding of the interactions between host and GCRV II.

## 1. Introduction

Grass carp reovirus (GCRV) is a double-stranded RNA (dsRNA) virus belonging to the genus *Aquareovirus* of the family *Reoviridae* [1]. GCRV infects a variety of fish and caused serious hemorrhage disease, resulting in huge economic losses to the aquaculture industry in China [2,3]. According to the VP6 protein sequence of GCRV, the known GCRV isolates were classified into three genotypes (Ⅰ–Ⅲ), and the sequence similarity between different genotypes was less than 20% [4,5]. A preliminary epidemiological analysis and detection of grass carp hemorrhagic disease fish collected from 2015 to 2017 in China showed that the positive rate of GCRV Ⅱ was as high as 89.8% [6], indicating that GCRV Ⅱ was the most common etiological agent of grass carp hemorrhagic disease. The mortality rates of grass carp caused by different GCRV Ⅱ strains were different. For example, the mortality rates of grass carp infected with HZ08, 109, and HuNan1307 strains were 30%, 80%, and 100%, respectively [7,8]. Investigating the pathogenic mechanism of GCRV Ⅱ is of great significance to improve the prevention and control of grass carp hemorrhagic diseases.

The rare minnow (*Gobiocypris rarus*) is a small cyprinid species endemic to China, with a total length of 3–6 cm and a short reproductive cycle. It is widely used in studies of ecotoxicology fields as a model fish [9]. In addition, rare minnow has been demonstrated to be sensitive to GCRV Ⅱ, making it an ideal model fish for research of GCRV Ⅱ pathogenesis [10,11]. In teleosts, the spleen is a primary hematopoietic and peripheral lymphoid organ [12] and is mainly responsible for microorganism defense, antigen presentation, and the start of adaptive immune responses [13,14]. The liver of teleosts serves as an important immune organ by housing numerous immune cell populations in addition to its functions in metabolism and the storing of nutrients [15]. Moreover, previous studies have shown that the liver and spleen are among the most severely infected tissues of grass carp and rare minnow [11,16,17,18].

Transcriptomic analysis can provide a comprehensive understanding of the intricate biological processes of fish response against infection at the transcriptome level [19,20]. With the rapid development of next-generation sequencing technology, a growing number of fish infectious diseases studies have been conducted using transcriptomics technology. For example, studies of Atlantic salmon infected with viruses [21], crucian carp and tilapia infected with bacteria [22,23], and large yellow croaker infected with parasites [24] have been reported. There are also several transcriptome studies conducted on GCRV-infected grass carp and rare minnow. He et al. [3] analyzed the kidney transcriptome of grass carp infected with GCRV Ⅰ and GCRV Ⅱ and found that mRNA expression of metabolism-related genes was downregulated and mRNA expression of immune-related genes was upregulated on the 5th day after GCRV Ⅱ infection, and the complement and coagulation cascade was the most enriched pathway. Chen et al. [25] studied the transcriptome of grass carp kidney cells (*Ctenopharyngodon idellus* kidney, CIK) infected with GCRV. It was found that there were three stages of infection: in the early stage (0–8 h), differentially expressed genes (DEGs) were mainly related to viral adhesion; in the middle stage (8–24 h), DEGs were mainly related to viral phagocytosis and transmission; in the late stage (24–72 h), DEGs were mainly concentrated in steroid metabolism that is important for membrane formation and apoptosis that involved in cell lysis. Lin et al. [26] studied the transcriptome of rare minnow infected with genotype Ⅱ virus GCRV-HZ08; when they compared it with the transcriptome of grass carp infected with GCRV-HZ08, they found that the responses of the two species were similar. Based on the above research results, GCRV Ⅱ infection may lead to host innate immune activation, metabolic dysfunction, and coagulation system disorders. However, the transcriptomic responses to virulent and attenuated GCRV Ⅱ infection have not been reported, as well as the difference in response between tissues has not been paid much attention in previous studies.

In the previous study, the experiments of mortality statistics, viral load measurement, and histological examination were conducted on rare minnow after attenuated GCRV Ⅱ isolate QJ205 and virulent GCRV Ⅱ isolate DY197 infection. The infection of QJ205 caused slightly muscular hemorrhage symptoms and 5% mortality in rare minnow, associated with low virus copy numbers and no obvious pathological changes in the spleen and liver. In contrast, DY197 infection led to severe muscular hemorrhage symptoms and 95% mortality in rare minnow, as well as approximately 100-fold virus copy numbers of those infected with attenuated QJ205 and severe cell necrosis in the spleen and liver [27]. To further dissect the virulence-specific and tissue-specific molecular mechanism, in the present study, we performed comparative transcriptome analysis in the spleen and liver of rare minnow after virulent and attenuated isolate infection. These results would undoubtedly help us to better understand the pathogenesis and host–pathogen interaction of GCRV Ⅱ infection.

## 2. Materials and Methods

### 2.1. Experimental Fish

Approximately 1500 healthy rare minnows weighing 1–1.5 g and measuring 4–4.5 cm were acquired from the Institute of Hydrobiology, Chinese Academy of Sciences (Wuhan, China). The fish were raised in a 500 L tank with a flow-through system and plenty of aeration at 28 °C prior to the experiment. The water was replaced every day, and the fish were fed twice daily. After ten days, if there were no abnormal symptoms, the virus challenge experiment was carried out.

### 2.2. Virus

The attenuated strain (GCRV-QJ205) and the virulent strain (GCRV-DY197) of GCRV Ⅱ were isolated from diseased grass carp that had been collected from the cities of Qianjiang in Hubei province and Deyang in Sichuan province, respectively. The spleens and livers of diseased grass carp were homogenized with a 5-fold amount of phosphate-buffered saline (PBS), and three freeze–thaw cycles at −80 °C were performed to isolate the virus. The tissue homogenate was then centrifuged for 30 min at 2880× *g*. The filtrate from the supernatant was diluted to a titer of 1 × 10^6^ RNA copies/L for the subsequent viral challenge experiment after being filtered through a 0.22 μm Millipore filter (Millipore, Billerica, MA, USA). 

### 2.3. Virus Infection and Sample Collecting 

Rare minnows were divided into three groups at random and given intraperitoneal injections of PBS (as a control group), QJ205, and DY197 at doses of 10 μL (1 × 10^6^ RNA copies/μL) for each fish. The samples (livers and spleens) of 480 and 9 fish from each group were collected at 5 days post-infection (dpi) and split into three parts as biological replicates for transcriptome sequencing and quantitative real-time PCR (qPCR) validation, respectively. The spleens and livers of the control, QJ205, and DY197 groups were designated as control-spleen (C-S), attenuated-spleen (A-S), virulent-spleen (V-S), control-liver (C-L), attenuated-liver (A-L), and virulent-liver (V-L), respectively.

### 2.4. RNA Extraction, cDNA Library Construction, and Sequencing

Using the Trizol reagent kit (Invitrogen, Carlsbad, CA, USA), the total RNAs were extracted from the spleen and liver samples in accordance with the manufacturer’s instructions. After the total RNA was isolated, Oligo(dT) beads were used to enrich mRNA. The short fragments created by using fragmentation buffer to break up the enriched mRNA were then reverse transcribed into complementary DNA (cDNA) using random primers. The second-strand cDNA fragments were then repaired at the terminal, A base was added and connected to the Illumina sequencing adapters. Agarose gel electrophoresis was used to size-select the ligation products, followed by PCR amplification and Illumina NovaSeq 6000 sequencing.

### 2.5. De Novo Assembly and Annotation

To get high-quality clean reads, the raw reads were further filtered by fastp (version 0.18.0). The parameters were as follows:

(1) removing reads containing adapters;
(2) removing reads containing more than 10% of unknown nucleotides (N);
(3) removing low-quality reads containing more than 50% of low-quality (Q-value ≤ 20) bases.

Subsequently, using the default settings in Trinity software [28], clean reads were de novo assembled and then were mapped back to the National Center for Biotechnology Information (NCBI) to remove GCRV Ⅱ contamination using the program Blast. Each cluster’s longest RNA was designated as a unigene. To annotate the unigenes, we used BLASTx software (http://www.ncbi.nlm.nih.gov/BLAST/, accessed on 1 June 2022) with an E-value threshold of 1 × 10^−5^ to NCBI non-redundant protein (Nr) database (http://www.ncbi.nlm.nih.gov, accessed on 1 June 2022), the COG/KOG database (http://www.ncbi.nlm.nih.gov/COG, accessed on 1 June 2022), the Kyoto Encyclopedia of Genes and Genomes (KEGG) database (http://www.genome.jp/kegg, accessed on 1 June 2022), and the Swiss-Prot protein database (http://www.expasy.ch/sprot, accessed on 1 June 2022). 

### 2.6. Gene Expression Analysis and Enrichment Analysis 

Gene expression levels were firstly estimated by mapping clean reads from each library back to the transcriptome assembly using Bowtie2 software [29] and then calculated read counts and normalized as FPKM (Fragments Per Kilobase of transcript per Millions mapped reads) values for each sample using RSEM software [30]. The DESeq R package [31] was used to carry out the differential analysis, and *p*-values were modified using Benjamini-Hochberg’s method. Finally, DEGs were designated as genes with an absolute value of log_2_ (Fold change) greater than 1 and an adjusted *p*-value less than 0.05. Then DEGs were enriched and analyzed with Gene Ontology (GO) term and KEGG pathway.

### 2.7. Validation of DEGs by RT-qPCR

Eight DEGs that were significantly differentially expressed in at least one isolate-infected group in each tissue were selected for the quantitative reverse transcript PCR (RT-qPCR) validation. As mentioned above, total RNA was extracted from spleen and liver tissue samples. The manufacturer’s instructions were followed to produce cDNA from the total RNA using a PrimeScript RT reagent Perfect Real Time Kit (TaKaRa, Dalian, China). Then, the reaction of qPCR was performed and analysed using a Rotor-Gene Q Series Software 1.7 supplied with the instrument (QIAGEN, Hilden, German). An amount of 10 μL of TB Green Premix Ex Taq Ⅱ (TaKaRa, Dalian, China), 2 μL of the cDNA sample, 0.8 μL (10 μM) of each primer, and ddH2O in a total volume of 20 μL made up the reaction mixtures. The reactions were amplified for 30 s at 95 °C, followed by 40 cycles of 95 °C for 10 s, 60 °C for 15 s, and 72 °C for 20 s.

Primer sequences of eight DEGs were listed in Table 1. For normalization of gene expression, *β-actin* gene was used as an internal control. Primers had a Tm of roughly 60 °C, and PCR products ranged in length from 100 to 200 bp. qPCR was conducted three times for each sample as technique replicates.

## 3. Results

### 3.1. Transcriptome Sequencing, De Novo Assembly, and Annotation

Transcriptomic sequencing generated totally 783 million raw reads from 18 libraries, which were deposited in the Sequence Read Archive (SRA) at the NCBI repository (accession number: PRJNA954066). After quality-filter analysis, 780 million clean reads were produced and de novo assembled into a total of 62,638 unigenes with an N50 length of 2385 bp (Table S1). All libraries gave Q20 ≥ 97%, Q30 ≥ 92%, and mapped percent ≥ 85% (Table S2). All unigenes were functionally annotated using four public databases, including Nr, Swiss-Prot, GO, and KEGG, for the assembled reference transcriptome. The results showed that 48.27% of unigenes were annotated by at least one of public database (Table S3). The Nr annotation demonstrated that 51.67% of unigenes in rare minnow liver and spleen could be annotated in the database of *Pimephales promelas* and *Anabarilius grahami* (Figure S1).

### 3.2. Identification and Enrichment of Differentially Expressed Genes

**Figure 1:**
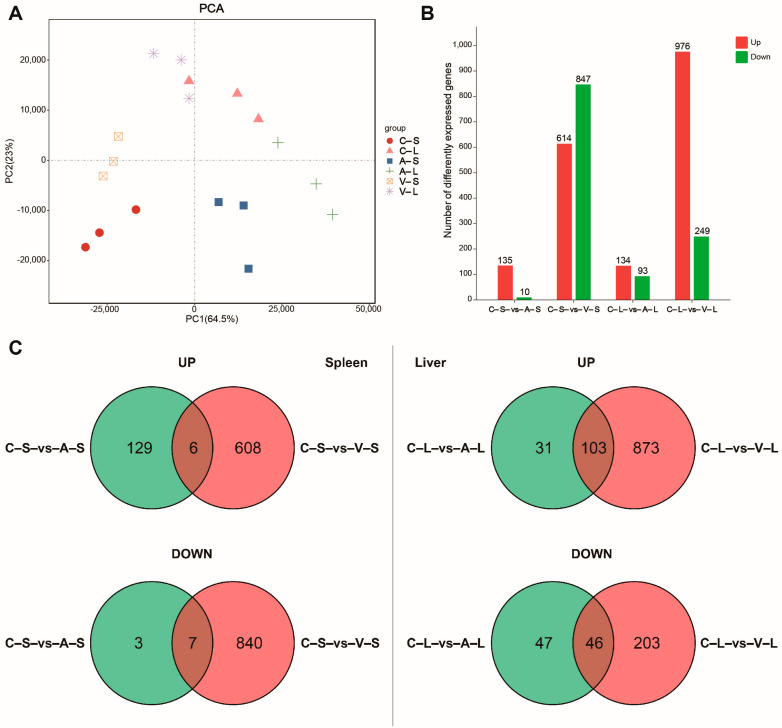
DEGs in rare minnow spleen and liver response to attenuated and virulent GCRV Ⅱ infection. (**A**) PCA score plot showing the difference in mRNA expression among the groups. (**B**) Barplot showing the summary of DEGs. (**C**) Venn diagrams showing the overlap of upregulated and downregulated DEGs in the attenuated and virulent GCRV Ⅱ infection groups. DEGs, differentially expressed genes; GCRV Ⅱ, grass carp reovirus genotype Ⅱ; PCA, principal component analysis; C–S, control–spleen; C–L, control–liver; A–S, attenuated–spleen; A–L, attenuated–liver; V–S, virulent–spleen; V–L, virulent–liver.

The PCA score plots showed good repeatability of the data, as the liver and spleen data sets were separated, while the same data set was clustered together (Figure 1A). These results demonstrated the sequencing data had high quality and was suitable for further investigation. To determine DEGs involved in response to GCRV Ⅱ infection in liver and spleen tissues of rare minnow, pairwise comparison for differential expression analysis was performed. In the spleen, compared with the control group, 145 DEGs (135 upregulated and 10 downregulated) were identified in the attenuated QJ205 infection group and 1461 DEGs (614 upregulated and 847 downregulated) in the virulent DY197 infection group (Figure 1B). In the liver, 227 DEGs (134 upregulated and 93 downregulated) were identified in the QJ205 infection group and 1461 DEGs (976 upregulated and 249 downregulated) in the DY197 infection group compared with the control group (Figure 1B). In both tissues, the virulent DY197 induced more DEGs than the attenuated QJ205, indicating the response to the infection of rare minnow was positively correlated with the virulence of GCRV Ⅱ. In the spleen, only six upregulated genes and seven downregulated genes were shared between the virulent and attenuated groups, indicating that the virulent and attenuated strain infection induced different responses (Figure 1C). In the liver, a total of 103 upregulated genes and 46 downregulated genes were shared in the virulent and attenuated strain infection groups, indicating that the virulent and attenuated strain infection induced similar responses in the liver (Figure 1C).

### 3.3. Enrichment Analysis of Differentially Expressed Genes in Spleen

**Figure 2:**
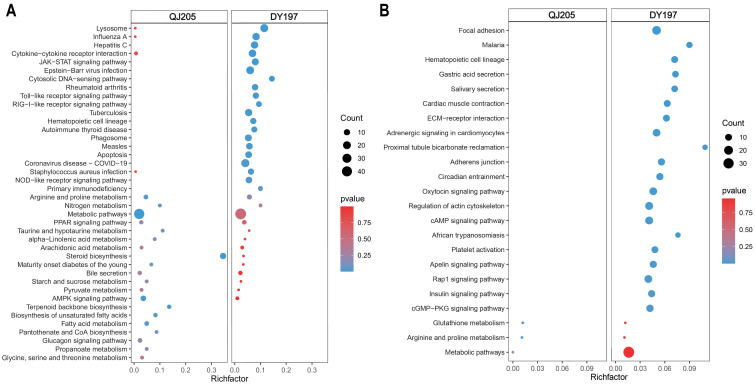
The top20 enrichment KEGG pathways of upregulated (**A**) and downregulated (**B**) differentially expressed genes in the spleen after infection with virulent and attenuated GCRV Ⅱ. GCRV Ⅱ, grass carp reovirus genotype Ⅱ; QJ205, attenuated strain of GCRV Ⅱ; DY197, virulent strain of GCRV Ⅱ. The comparisons were between the infected group versus the control group.

In the spleen, KEGG enrichment analysis (Figure 2) showed different pathways were induced in both infected groups. Among the upregulated pathways, innate immunity-related pathways, such as RIG-Ⅰ, TOLL, Nod-like receptor signaling pathways, JAK-STAT signaling pathway, lysosome, phagosome, and apoptosis, were significantly enriched in the virulent DY197 infection group. Lipid metabolic pathways, such as steroid metabolism, biosynthesis of unsaturated fatty acid, and fatty acid metabolism, were significantly enriched in the attenuated QJ205 infection group. In the downregulated pathways, cell migration and proliferation-related pathways such as focal adhesion, extracellular matrix receptor interaction (ECM-receptor interaction), regulation of actin cytoskeleton pathways, adaptive immunity-related pathways such as hematopoietic cell lineage, T cell receptor signaling pathway, and hemorrhage-related pathways such as malaria and platelet activation were significantly enriched in the DY197 infection group. In addition, as shown in Table 2, the expression levels of DEGs in these pathways changed more significantly in the virulent DY197 infection group.

### 3.4. Enrichment Analysis of Differentially Expressed Genes in Liver

**Figure 3:**
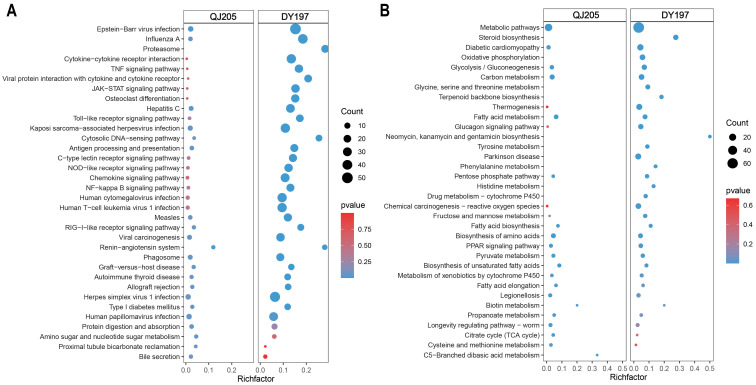
The top 20 enrichment KEGG pathways of upregulated (**A**) and downregulated (**B**) differentially expressed genes in the liver after infection with virulent and attenuated GCRV Ⅱ. GCRV Ⅱ, grass carp reovirus genotype Ⅱ; QJ205, attenuated strain of GCRV Ⅱ; DY197, virulent strain of GCRV Ⅱ. The comparisons were between the infected group versus the control group.

In the liver, KEGG enrichment analysis (Figure 3) showed that similar pathways were induced in both infected groups. Immunity pathways and protein digestion and absorption pathway were significantly activated after both strains of infection; the proteasome pathway was only significantly enriched in the virulent DY197 infection group; while metabolic pathways were inhibited, and the steroid synthesis pathway was only enriched in the virulent DY197 infection group. As shown in Table 3, virulent DY197 infection not only induced more DEGs but also induced greater changes in the expression level of DEGs.

### 3.5. Validation of Differentially Expressed Genes by qPCR

**Figure 4:**
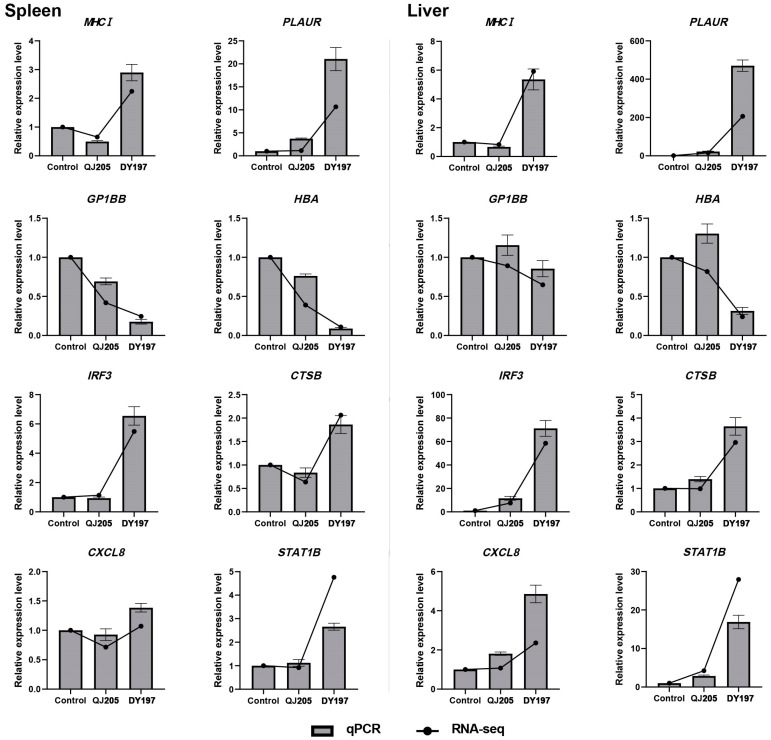
Validation of differentially expressed genes in RNA-seq data by using qPCR.

Eight DEGs involved in the immune-related pathways, lysosome pathway, and hemorrhage-related pathways were selected for qPCR validation. These eight DEGs included MHC class I antigen (*MHCI*), interferon regulatory factor 3 (*IRF3*), C-X-C motif chemokine 8 (*CXCL8*), signal transducer and activator of transcription 1b (*STAT1B*), cathepsin B (*CTSB*), urokinase plasminogen activator surface receptor (*PLAUR*), hemoglobin subunit alpha (*HBA*), and platelet glycoprotein Ib beta chain (*GP1BB*). As shown in Figure 4, qPCR expression trends of these eight DEGs were consistent with transcriptome results, which confirmed the accuracy and reliability of RNA-seq results.

## 4. Discussion

**Figure 5:**
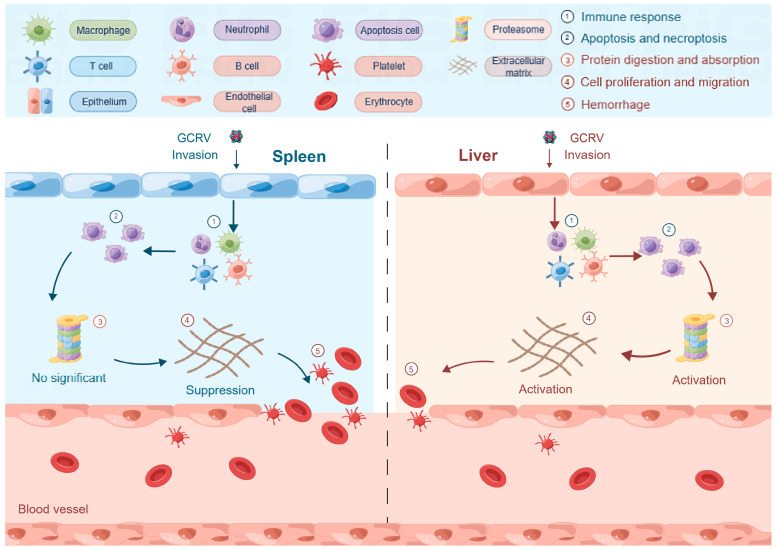
Schematic diagram showing the different regulatory mechanisms in the spleen and liver of rare minnow after GCRV Ⅱ infection from the results of this study. (By Figdraw.). When rare minnow was infected with GCRV Ⅱ, immune response and apoptosis-related pathways were activated in the spleen and liver because GCRV Ⅱ infection especially activated protein digestion and absorption-related pathways in the liver only, cell migration and proliferation-related pathways that are important for tissue repair were activated by sufficient protein supply in the liver but suppressed in the spleen; eventually, hemorrhage was relatively mild in the liver but severe in the spleen.

GCRV Ⅱ causes severe hemorrhagic disease in grass carp and affects the aquaculture industry in China. Previous studies have demonstrated that GCRV Ⅱ infection induced innate immunity activation, metabolic dysfunction, and coagulation disorder. However, the underlying virulence-specific and tissue-specific pathogenesis of GCRV Ⅱ infection remains to be further studied. For this reason, we performed a comparative transcriptomic analysis in the spleen and liver of rare minnow injected with virulent isolate DY197 and attenuated isolate QJ205 to investigate the possible involved molecular responses against the GCRV Ⅱ infection. The results showed the number of DEGs was positively correlated with the virulence of GCRV Ⅱ. In the spleen, compared with attenuated QJ205 infection, virulent DY197 infection activated innate immunity and apoptosis-related pathways but suppressed adaptive immunity, cell proliferation and migration, and hemorrhage-related pathways. In the liver, except innate immunity and apoptosis-related pathways, virulent DY197 infection especially activated protein digestion and absorption-related pathways, both innate and adaptive immunity-related pathways, and cell migration and proliferation-related pathways and caused slight suppression of hemorrhage-related pathways. The different regulatory mechanisms in the spleen and liver after GCRV Ⅱ infection were shown in Figure 5.

### 4.1. Immune Response

As pattern recognition receptors (PRRs), RIG-I, Toll, and Nod-like receptors can recognize the unique pathogen-associated molecular pattern (PAMPs) or damage-associated molecular pattern (DAMPs) components of the organism and initiate downstream inflammatory responses in response to infection when the host is infected with a pathogen [32]. Extracellular cytokines can be recognized by corresponding receptors, stimulate the JAK-STAT pathway, regulate transmembrane receptor communication to the nucleus, and promote the expression of related antiviral genes [33]. However, the activation of STAT also causes tissue damage and leads to hemorrhage [34,35]. Members of the cytokine signal transduction inhibitors (SOCS) family are key regulators of immune balance [36,37]. In this study, in the spleen of virulent DY197-infected group, the mRNA expression levels of Toll-like receptor 3 (*TLR3*) and Toll-like receptor 8 (*TLR8*) in the Toll-like receptor signaling pathway; interferon regulatory factor 3 (*IRF3*), interferon regulatory factor 7 (*IRF7*), interferon (*IFN-α* and *IFN-β*), Retinoic acid-inducible protein I (*RIG-I*) in RIG-I receptor pathway; melanoma differentiation associated gene 5 (*MDA5*), DEXH-box polypeptide 58 (*LGP2*), *JAK1* and *STAT1B* in JAK-STAT signaling pathway were significantly upregulated, enhancing inflammation to restrict viral infection, while mRNA expression levels of *SOCS1*, *SOCS3* and *SOCS6* were upregulated, controlling inflammation and avoid excessive damage. This regulatory mode was also observed in the liver of the virulent DY197 infection group. In comparison, the mRNA expression levels of related genes were slightly upregulated or even downregulated in the spleen and liver of the attenuated QJ205-infected group.

Phagocytosis of macrophages and degradation of lysosomes are the last step of phagocytosis and elimination of microorganisms [38]. For example, as a protease of lysosomes, cathepsin L (CTSL) is involved in a variety of immune responses, including apoptosis, antigen presentation, and inflammation [39,40]. In this study, as important effecting genes and antigen presentation genes in the lysosome and phagosome pathways, mRNA expression levels of cathepsins (*CTSL*, *CTSS*, *CTSZ*, *CTSA*, *CTSK*, and *CTSB*) and major histocompatibility complex class Ⅰ antigen (*MHC Ⅰ*) were significantly upregulated in both virulent DY197 infected liver and spleen and slightly upregulated or even downregulated in attenuated QJ205 infected liver and spleen, indicating that the virulent DY197 infection activated phagocytosis of macrophages. Similar to our study, the activation of phagosome and lysosome pathways was also found in the spleen and kidney of GCRV Ⅱ-infected grass carp [3,41].

However, in terms of adaptive immunity, the B cell receptor signaling pathway was activated in both liver and spleen, while the T cell receptor signaling pathway behaved differently in the liver and spleen. CD8 cell surface receptors *CD8A* and *CD3Z* genes were downregulated in the spleen, while *CD3Z* genes were upregulated in the liver. Previous studies in human viruses, such as herpes simplex virus (HSV) and SARS coronavirus 2 (SAR-COV-2), have demonstrated that activation of the kynurenine pathway of tryptophan metabolism can enhance antioxidant to alleviate inflammation in innate immune cells, but depletion of tryptophan will lead to suppression of T cell [42,43]. In this study, kynurenine-oxoglutarate transaminase 1 (*KYAT1*), the kynurenine pathway gene of tryptophan metabolism, was significantly upregulated in both spleen and liver after the virulent DY197 infection (Table 2 and Table 3). T cell was suppressed in the spleen and activated in the liver, which may be related to a sufficient supply of tryptophan because of liver-specific protein synthesis and metabolism function as discussed below. Tissue-specific inhibition of T cells provides us new insight into further investigation of the pathogenesis of GCRV Ⅱ infection.

### 4.2. Apoptosis and Necroptosis-Related Pathways

Apoptosis and necroptosis can be activated through stimulation of IFN and tumor necrosis factor (TNF), DNA damage, depletion of cellular NAD+, production of iron-dependent ROS, mitochondrial permeability change, etc. [44]. IFN transcription activates RNA reactive protein kinase, triggering necrosis through JAK1–STAT1-dependent transcription [45]. TNF stimulates the production of caspase-7/8, which plays an important role in the pathogen clearance and apoptosis of damaged cells [46]. DNA damage causes mitochondrial apoptosis induced by BAX (Bcl-2-associated X protein) [47]. Transcriptomic studies of GCRV-infected CIK [25] showed that necroptosis and apoptosis pathways were activated, and the steroid synthesis pathway that involved cell membrane formation [48] was suppressed in the later stage of infection. In this study, after virulent DY197 infection, the mRNA expression levels of tumor necrosis factor receptor superfamily member 1A (*TNFRSF1A*), tumor necrosis factor superfamily member 10 (*TNFSF10*), *caspase-7*, *caspase-8*, *BAX* genes were significantly upregulated in the liver, and the mRNA expression levels of *caspase7* and *BAX* genes were significantly upregulated in the spleen; while the mRNA expression levels of these genes were slightly upregulated after attenuated QJ205 infection in both liver and spleen. In addition, the steroid synthesis pathway associated with membrane formation was suppressed in both spleen and liver after the virulent DY197 infection, which was consistent with the virulent DY197 infection that caused severe cell necrosis and tissue damage observed by histological examination [27]. Interestingly, the steroid synthesis pathway was activated in the spleen of attenuated QJ205 infected rare minnow, which may indicate that the spleen had passed the peak of antiviral response and began to synthesize steroids to restore membrane formation at 5 dpi. Previous studies have shown that hemorrhage symptoms gradually appeared on the 5th to 7th day in the head kidney of grass carp after a virulent strain of GCRV Ⅱ infection, and the upregulation of immune gene expression and downregulation of metabolic gene expression caused by viral infection would be reversed on the 7th day [3]. This reversal may be a regulatory mechanism for recovery after viral infection. Another study on viral load of GCRV Ⅱ-infected grass carp showed spleen and kidney were the tissues with the first increase in viral load and reached the peak of viral load on the 5th day, which may be due to the accumulation of virus caused by immune, hematopoietic, or glomerular filtration in these two tissues [16,17]. In studies of rare minnow infected with a virulent isolate of GCRV Ⅱ, the spleen was also the tissue that reached the viral load peak relatively quickly [11]. Considering GCRV Ⅱ tends to replicate faster and cause higher mortality in rare minnow than in grass carp after GCRV Ⅱ infection [11,16], it is possible that the spleen has passed the peak of disease and started to recover in rare minnow on the 5th day after attenuated QJ205 infection.

### 4.3. Protein Digestion and Absorption

The protein digestion and absorption pathway was activated only in the liver after both strain infections. Solute carrier family 15 member 1 (*SLC15A1*) chiefly mediates di/tripeptides absorption from protein digestion [49]. Meprin alpha, a zinc metalloprotease, was previously reported to be capable of cleaving a variety of substrates (e.g., protein kinases, basement membrane proteins, cytokines), and participate in the regulation of fibroblast activation and production of extracellular matrix [50]. In this study, the mRNA expression of meprin alpha subunit A/B (*MEP1A*, *MEP1B*) and *SLC15A1* genes was significantly upregulated after both strain infections in the liver. The proteasome can degrade a large number of damaged and misfolded proteins, and then the production can be used to synthesize new proteins required by the organism. Thus, the proteasome controls many biological processes, including cell cycle, cell survival, and apoptosis [51,52]. In this study, in the liver, the proteasome pathway was the most significantly different pathway between the virulent DY197 and attenuated QJ205 infection group. In contrast, in the spleen, only two proteasome genes were significantly upregulated after virulent DY197 infection, and no related genes were upregulated after attenuated QJ205 infection. 

The activation of protein digestion and absorption and proteasome pathways induced by GCRV Ⅱ infection may lead to an increase in oligopeptides and amino acids of proteolytic products, which was consistent with protein synthesis and metabolism for liver-specific function. The tissue-specific activation of protein digestion and absorption and proteasome pathways may be responsible for T cell activation in the liver but suppression in the spleen after virulent DY197 infection as described above. In addition, the lack of protein digestion and absorption and proteasome activation may result in the inhibition of many biological processes controlled by these two pathways in the spleen after virulent DY197 infection, including cell cycle, cell survival, and apoptosis [51,52].

### 4.4. Cell Proliferation and Migration

Focal adhesion is a subcellular structure that regulates the adhesion response of cells to the extracellular matrix [53]. Focal adhesion kinase is the core of the focal adhesion pathway; it can communicate with integrin, vascular endothelial growth factor receptor (VEGFR), platelet-derived growth factor receptor (PDGFR), insulin-like growth factor receptor (IGFR) and actin-related proteins that interact with each other to regulate cell proliferation, migration, and survival [54,55]. The regulation of the actin cytoskeleton pathway is downstream of the focal adhesion pathway and is involved in the regulation of cell movement. Previous studies have shown that focal adhesion kinase can interact with phosphoproteins of rabies virus and participate in viral infection [56]; during infection of shrimp, white spot syndrome virus was also found to interact with integrin proteins of the focal adhesion pathway [57]. Studies of GCRV-infected CIK, kidney, and spleen of grass carp showed that the focal adhesion and regulation of actin cytoskeleton pathways were activated at the initial stage of infection, suggesting these two pathways may be involved in the binding of the virus to the receptor; while these two pathways were suppressed at the later stage of infection, suggesting that the host translation mechanism was hijacked or shut down to promote virus replication and transmission [3,25,41]. In this study, after virulent DY197 infection, the mRNA expression of platelet-derived growth factor C (*VEGFC*), platelet-derived growth factor receptor subunit B (*PDGFB*), integrin alpha-2 (*ITGA2*), myosin regulatory light polypeptide 9b (*MYL9*), myosin light chain kinase (*MYLK*) genes involved in focal adhesion and regulation of actin cytoskeleton pathways were significantly downregulated in the spleen, which may cause cell proliferation and migration disorders that are important for repairing of tissue damage. The damage to vascular endothelial cells can lead to hemorrhage. In comparison, attenuated QJ205 infection induced only slight downregulation of mRNA expression of related genes in the spleen. Our spleen results were similar to previous transcriptome studies on the spleen of grass carp infected with GCRV Ⅱ [41]. However, our data suggested that the focal adhesion and regulation of actin cytoskeleton pathways were activated in the liver after GCRV Ⅱ infection, which had not been reported. Such tissue-specific regulatory mode of these two pathways may be controlled by metabolite abundance, as activation of protein digestion and absorption and proteasome pathways was liver-specific function.

### 4.5. Hemorrhage-Related Pathways

The complement and coagulation cascade pathway was activated in the spleen and liver after the virulent DY197 infection. In comparison, attenuated QJ205 infection induced only slight changes in the expression of genes involved in this pathway. The complement and coagulation cascade system has been reported to play an important role in innate immunity [58,59]. Overactivity of the complement cascade, however, can lead to endothelial damage, platelet activation and aggregation, hemolysis, and thrombosis [60,61]. Thus, significant activation of the complement and coagulation cascade pathway may account for the hemorrhagic symptoms after virulent DY197 infection. Malaria is an infectious disease caused by plasmodium, which causes a decrease in hemoglobin in human blood [62]. In this study, the mRNA expression of hemoglobin subunit alpha (*HBA*), hemoglobin subunit beta (*HBB*), thrombospondin-1 (*THBS1)*, platelet glycoprotein Ib beta chain (*GP1BB*) genes involved in malaria and platelet activation pathways were significantly downregulated in the spleen after the virulent DY197 infection. In comparison, the mRNA expression of these genes was relatively slightly downregulated in the liver after the virulent DY197 infection. Obviously, although GCRV Ⅱ infection in the liver activated the complement and coagulation cascade system, due to the activation of proteasome, focal adhesion, and regulation of actin cytoskeleton pathways, vascular endothelial cells in the liver may be actively repaired after damage, alleviating hemorrhage symptoms. 

## 5. Conclusions

In this study, a comparative transcriptomic analysis in the spleen and liver of rare minnow injected with virulent strain DY197 and attenuated strain QJ205 was conducted to investigate the possible involved molecular responses against the GCRV Ⅱ infection. The results showed the virulent DY197 strain induced more DEGs than the attenuated QJ205 strain, and tissue-specific responses were induced. In the spleen, virulent DY197 infection activated innate immunity and apoptosis-related pathways but suppressed adaptive immunity, cell migration and proliferation, and hemorrhage-related pathways. In the liver, except innate immunity and apoptosis-related pathways, virulent DY197 infection especially activated protein digestion and absorption-related pathways, both innate and adaptive immunity-related pathways, cell proliferation and migration-related pathways and caused slight suppression of hemorrhage-related pathways. These results would help us to better understand the interactions between the host and GCRV II.

## Figures and Tables

**Table 1:** The primers of differentially expressed genes.

Gene Name	Forward Primer (5′-3′)	Reverse Primer (5′-3′)
*β-actin*	ATGGATGATGAAATTGCCGC	CTGTCCCATACCAACCATGA
*GP1BB*	GAACTCCACCTGAACGACAA	TCAGATAAAGGATGCCGCAG
*MHC* *Ⅰ*	CCTATGCTGGACAACACTCT	TTTGGCACAGCTTTCATTGC
*PLAUR*	TAACGTGCCCAATGGAAAGA	TACAACCAAAGACTGGCCTC
*IRF3*	TCAGTGGGAAGATCAACGAG	TGAGGACGGATAATGCGAAA
*CTSB*	CTGGCTACAGCCCTTCTTAC	AGGAAGTCCTCATACACGGT
*CXCL8*	CAGTCTTTGTTATCGCTGGC	ATGTGTTTACCAATGCGTCG
*HBA*	AGACCTATTTCGCTCACTGG	GAATGCATGAAGTTCGCTCA
*STAT1B*	AGCACTACAGCCGTCTCAATCT	CCTGTGATGAGTTACCGCTACCTT

**Table 2:** Representative differentially expressed genes in the spleen of attenuated QJ205 and virulent DY197 infected groups. The log_2_ (Fold change) was calculated using the formula log_2_ (infected group/control group).

Pathway	Gene Name	Log_2_ (Fold Change)	
		QJ205	DY197
Representative upregulated differentially expressed genes			
RIG-Ⅰ/TOLL/NOD-like receptor signaling pathway, JAK-STAT signaling pathway	Retinoic acid-inducible protein I	0.11	2.01
	DEXH (Asp-Glu-X-His) box polypeptide 58	0.28	2.47
	Melanoma differentiation associated gene 5	−0.07	1.72
	Toll-like receptor 3	−0.17	1.03
	Toll-like receptor 8	−0.97	1.02
	Interferon regulatory factor 3	0.09	1.06
	Interferon regulatory factor 7	0.16	2.44
	Interferon alpha	−0.81	1.60
	Interferon beta	−0.56	3.85
	Signal transducer and activator of transcription 1b	−0.13	2.24
	Signal transducer and activator of transcription 2	−0.34	1.09
	Tyrosine-protein kinase JAK1	−0.61	1.27
	Chemokine (C-X-C motif) ligand 11	−0.37	1.79
	Chemokine (C-X-C motif) ligand 20	−0.11	2.23
	Suppressor of cytokine signaling 1	0.08	2.06
	Suppressor of cytokine signaling 3	−0.44	1.18
	Suppressor of cytokine signaling 6	−0.84	1.38
B cell receptor signaling pathway	Immunoglobulin omega chain	−0.70	1.96
	B-cell receptor CD22	−1.03	1.05
	Secreted immunoglobulin heavy chain IgM	−0.73	1.16
	Immunoglobulin heavy variable 1–3	−0.59	1.81
Tryptophan metabolism	kynurenine-oxoglutarate transaminase 1	−0.62	1.34
Lysosome and phagosome	Cathepsin A	−0.69	1.10
	Cathepsin B	−0.65	1.05
	Cathepsin D	−0.82	1.17
	Cathepsin K	−0.30	2.33
	Cathepsin L	0.16	1.83
	Cathepsin S	−0.71	1.06
	Cathepsin Z	−0.64	1.08
	MHC class I antigen	−0.61	1.17
Apoptosis and necroptosis	Bcl-2 associated X protein	−0.19	1.64
	Caspase-7	−0.66	1.81
	Interferon-induced, double-stranded RNA-activated protein kinase	0.10	1.56
Complement and coagulation cascades	Coagulation factor IXa	1.05	0.21
	Urokinase plasminogen activator surface receptor	0.18	3.41
	Complement factor H	0.58	1.48
Representative downregulated differentially expressed genes			
T cell receptor signaling pathway	T-cell surface glycoprotein CD3 zeta chain	−1.26	−1.62
	CD8 alpha chain	−0.93	−2.17
	Tyrosine-protein kinase ZAP-70	−0.63	−1.45
Steroid biosynthesis	Squalene synthase	1.12	−0.48
	Lanosterol synthase	1.39	−1.50
	Delta(24)-sterol reductase	1.37	−0.77
	Squalene monooxygenase	1.86	−2.10
Malaria	Hemoglobin subunit alpha	−1.36	−3.21
	Hemoglobin subunit beta-2	−0.33	−1.98
	Thrombospondin-1	−1.08	−1.79
Platelet activation	Platelet glycoprotein V	−1.15	−1.96
	Platelet glycoprotein Ib alpha chain	−0.95	−1.80
	Platelet glycoprotein Ib beta chain	−1.26	−2.04
Focal adhesion and regulation of actin cytoskeleton	Myosin regulatory light polypeptide 9b	−1.31	−1.67
	Myosin light chain kinase	−0.92	−1.62
	Integrin alpha-2	−1.09	−1.66
	Platelet-derived growth factor subunit B	−1.42	−2.06
	Laminin subunit beta-1b	−0.69	−1.67
	Vascular endothelial growth factor C	−1.07	−1.48

**Table 3:** Representative differentially expressed genes in the liver of attenuated QJ205 and virulent DY197 infected groups. The log_2_ (Fold change) was calculated using the formula log_2_ (infected group/control group).

Pathway	Gene Name	Log_2_ (Fold Change)	
		QJ205	DY197
Representative upregulated differentially expressed genes			
RIG-Ⅰ/TOLL/NOD-like receptor singaling pathway, JAK-STAT singaling pathway	Retinoic acid-inducible protein I	1.95	3.99
	DEXH (Asp-Glu-X-His) box polypeptide 58	2.29	4.70
	Melanoma differentiation associated gene 5	0.93	3.13
	Nucleotide-binding oligomerization domain-containing protein	0.02	1.67
	Nicotinamide phosphoribosyltransferase-like protein	1.05	3.54
	Toll-like receptor 8	0.52	3.10
	TNF receptor-associated factor 5	0.16	2.47
	Interferon regulatory factor 7	2.85	5.77
	Interferon regulatory factor 9	0.56	2.49
	Interferon a	0.71	3.86
	Signal transducer and activator of transcription 1b	2.08	4.79
	Tyrosine-protein kinase JAK1	−0.04	1.74
	Cytokine receptor family member b4	−0.03	1.96
	C-X-C motif chemokine 9	1.10	3.90
	Chemokine (C-X-C motif) ligand 20	0.56	4.47
	Suppressor of cytokine signaling 1	0.87	2.52
	Suppressor of cytokine signaling 3	0.21	1.79
B cell receptor signaling pathway	Immunoglobulin omega chain	0.00	8.06
	B-cell receptor CD22	0.58	2.81
	Secreted immunoglobulin heavy chain IgM	0.73	2.08
Tryptophan metabolism	kynurenine-oxoglutarate transaminase 1	0.61	1.81
Lysosome and phagosome	Cathepsin A	−0.05	1.15
	Cathepsin B	−0.02	1.56
	Cathepsin H	0.27	1.81
	Cathepsin K	1.19	3.87
	Cathepsin L	0.07	1.53
	Cathepsin Z	−0.12	1.82
	MHC class I antigen	0.20	3.28
Apoptosis and necroptosis	Bcl-2 associated X protein	−0.19	1.64
	Caspase-7	0.29	2.69
	Caspase-8	1.65	3.75
	Interferon-induced, double-stranded RNA-activated protein kinase	1.16	2.80
	Tumor necrosis factor receptor superfamily member 1A	0.24	1.98
	Tumor necrosis factor (ligand) superfamily, member 10	1.94	3.00
Proteasome	Proteasome subunit alpha type-3	0.10	1.50
	Proteasome subunit alpha type-5	−0.06	1.27
	Proteasome subunit alpha type-6	0.51	2.25
	Proteasome subunit beta type-2	−0.33	1.36
	Proteasome subunit beta type-7	0.99	2.97
	Proteasome subunit beta type-8	0.54	2.97
	Proteasome activator complex subunit 1	0.38	2.33
	Proteasome activator complex subunit 2	0.46	2.81
Protein digestion and absorption	Meprin A subunit alpha	2.14	3.49
	Meprin A subunit beta	2.80	3.19
	Solute carrier family 15 member 1	3.26	3.05
Complement and coagulation cascades	Complement C1q subcomponent subunit A	0.51	2.49
	Proteinase-activated receptor 1	−0.08	1.79
	Urokinase plasminogen activator surface receptor	3.96	7.69
	Complement factor H	−0.19	1.44
Representative downregulated differentially expressed genes			
Steroid biosynthesis	Squalene synthase	−0.24	−1.52
	Lanosterol synthase-like isoform X2	−0.75	−2.02
	Delta(24)-sterol reductase	−0.02	−1.14
	Squalene monooxygenase	−0.98	−2.59

## Data Availability

The data presented in this study are available in the article or supplementary material.

## Supplementary Materials

The following supporting information can be downloaded at: https://www.mdpi.com/article/10.3390/ani13111870/s1, Table S1: The summary of de novo assembly of transcriptomic profiles of rare minnow; Table S2: Summary statistics of sequencing data in the transcriptomes of rare minnow; Table S3: Summary of unigenes annotation of the spleen and liver of rare minnow. Figure S1: The Nr annotation summary of the de novo assembled transcriptome of rare minnow spleen and liver.
