# Supplementary material for: Transcriptomics in Rare Minnow (Gobiocypris rarus) towards Attenuated and Virulent Grass Carp Reovirus Genotype II Infection
Source: Animals (Basel). 2023 Jun 4;13(11):1870. doi: 10.3390/ani13111870 (PMC10251909; doi:10.3390/ani13111870)
Supplement: Supplementary file 1 [file animals-13-01870-s001.zip › Figure S1.pdf]

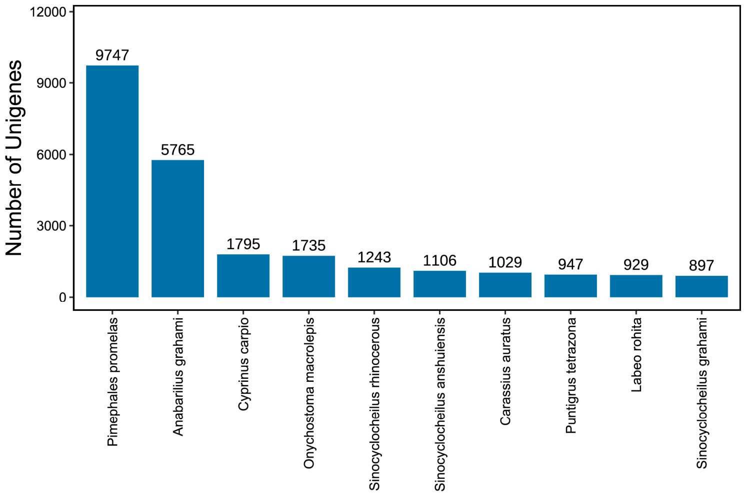

Figure S1. The Nr annotation summary of the *de novo* assembled transcriptome of rare minnow spleen and liver.
